# Phylogenetic relationship and characterization of the complete mitochondrial genome of the black citrus aphid, *Aphis aurantii* (Hemiptera: Aphididae)

**DOI:** 10.1080/23802359.2019.1674208

**Published:** 2019-10-15

**Authors:** Ying Wang, Min Ding, Yimin Du, Aijun Huang

**Affiliations:** a School of Life Sciences, Gannan Normal University, Ganzhou, Jiangxi, China;; b National Navel Orange Engineering and Technology Research Center, Ganzhou, Jiangxi, China

**Keywords:** Aphididae, mitochondrial genome, *Aphis aurantii*, phylogenetic analysis

## Abstract

The black citrus aphid *Aphis aurantii* is a major pest of citrus and tea plants. In this study, we determined the complete mitochondrial genome (mitogenome) of *A. aurantii*. The mitogenome was 15,469 bp in length, consisting of 13 protein-coding genes (PCGs), 22 transfer RNA (tRNA) genes, 2 ribosomal RNA (rRNA) genes, and a non-coding control region. Gene order of *A. aurantii* was highly conserved and identical to most other previously sequenced Aphididae. Initiation codons ATT, ATA, and ATG were identified in eight, three, and two PCGs, respectively, while stop codon TAA was found in 11 genes except for 2 genes (*cox1* and *nad4*) which use incomplete stop codon T–. Phylogenetic analysis showed the topology (Pemphigidae + (Hormaphididae + (Greenideidae + Aphididae))) within Aphidoidea, and *A. aurantii* was more closely related to *Aphis craccivora* than to *Aphis gossypii*.

The black citrus aphid, *Aphis* (*Toxoptera*) *aurantii* (Boyer de Fonscolombe) (Hemiptera: Aphididae), is a polyphagous species with a worldwide distribution (Tahori and Hazan 1970; Wang and Tsai 2001). It has over 120 hosts but shows a preference for members of the Rutaceae, Rosaceae, Apocynaceae, and Rubiaceae (Carver 1978). *Aphis aurantii* has been considered as one of the most destructive pests of citrus and tea plants in some areas (Carver 1978; Sevim et al. 2012).

Specimens of *A. aurantii* were collected from Hezhou City, Guangxi Province, China (24°18′N, 111°24′E, May 2019) and were stored in Entomological Museum of Gannan Normal University (Accession number GNU-AP058). After morphological identification, total genomic DNA was extracted from tissues using the DNeasy DNA Extraction kit (Qiagen, Hilden, Germany). Mitogenome sequence was generated using Illumina HiSeq Sequencing System (Illumina, San Diego, CA). In total, 5.2 G raw reads were obtained, quality-trimmed and assembled using MITObim v 1.7 (Hahn et al. 2013). By comparison with the homologous sequences of other Aphididae species from GenBank, the mitogenome of *A. aurantii* was annotated using software GENEIOUS R8 (Biomatters Ltd., Auckland, New Zealand).

The complete mitogenome of *A. aurantii* is 15,469 bp (Genbank accession, MN397939). It contains 13 protein-coding genes (PCGs), 22 tRNA genes, 2 rRNA genes, and a non-coding AT-rich region. Most mitochondrial genes were encoded on the majority strand except for four PCGs, two rRNA genes, and eight tRNA genes. The gene order of *A. aurantii* was highly conserved and identical to most other previously sequenced Aphididae (Thao et al. 2004; Ren et al. 2016; Li et al. 2017; Chen et al. 2019). The overall base composition was A (44.7%), T (38.8%), C (10.6%), and G (5.9%), with an AT content of 83.5%, which was consistent with most mitochondrial genomes. All PCGs started with the standard ATN codons (eight ATT, three ATA, and two ATG). Most of the PCGs terminated with the TAA stop codon, while *cox1* and *nad4* end with the incomplete codon T. Two rRNA genes (*rrnL* and *rrnS*) located at *trnL1*/*trnV* and *trnV*/control regions, respectively. The lengths of *rrnL* and *rrnS* in *A. aurantii* were 1260 and 776 bp, with the A + T contents of 84.8 and 84.4%, respectively. The 22 tRNA genes vary from 62 bp (*trnW*, *trnS1*, *trnT*) to 73 bp (*trnK*).

All 13 mitochondrial protein-coding genes sequences were extracted from the mitochondrial DNA sequences of 18 closely related taxa of Aphidoidea, including one outgroup species from Phylloxeroidea. A phylogenetic tree was constructed using the maximum-likelihood method through raxmlGUI 1.5 (Silvestro and Michalak 2012). Results showed that within Aphidoidea, the topology (Pemphigidae + (Hormaphididae + (Greenideidae + Aphididae))) was recovered (Figure 1). *Aphis* had a close relationship with *Schizaphis* and *Rhopalosiphum*, and the newly sequenced species *A. aurantii* was more closely related to *Aphis craccivora* than to *Aphis gossypii*. In conclusion, the complete mitochondrial genome sequence of *A. aurantii* provides an important molecular framework for further phylogenetic analyses of Aphidoidea.

## Figures and Tables

**Figure 1.**
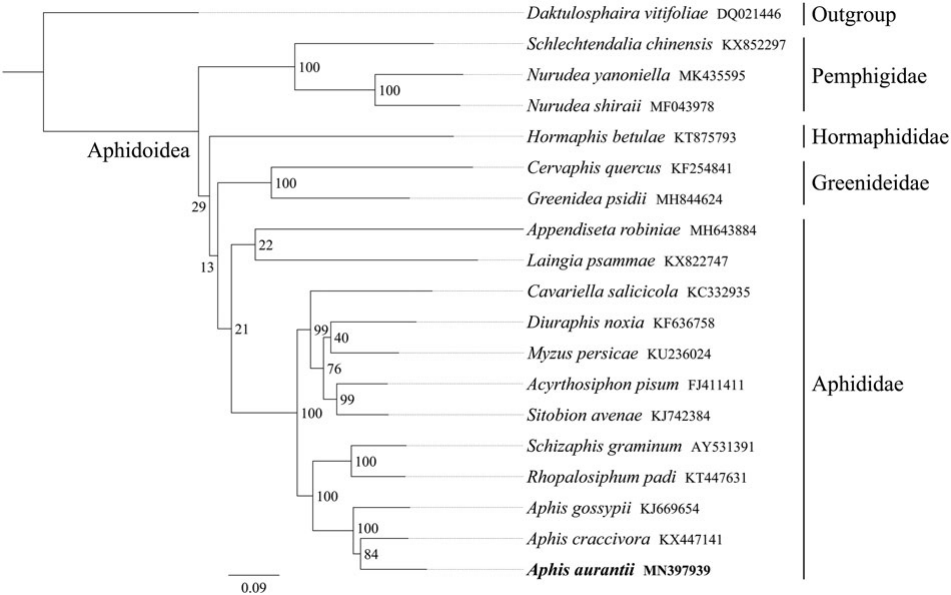
Phylogenetic relationships based on the 13 mitochondrial protein-coding genes sequences inferred from RaxML. Numbers on branches are bootstrap support values (BS).
